# The role of gender power relations on women’s health outcomes: evidence from a maternal health coverage survey in Simiyu region, Tanzania

**DOI:** 10.1186/s12889-021-10972-w

**Published:** 2021-05-13

**Authors:** Henri M. Garrison-Desany, Emily Wilson, Melinda Munos, Talata Sawadogo-Lewis, Abdoulaye Maïga, Onome Ako, Serafina Mkuwa, Amy J. Hobbs, Rosemary Morgan

**Affiliations:** 1 Department of Epidemiology, Johns Hopkins Bloomberg School of Public Health, 615 North Wolfe St, Baltimore, MD 21205 USA; 2 Department of International Health, Johns Hopkins Bloomberg School of Public Health, 615 North Wolfe St, Baltimore, MD 21205 USA; 3 Amref Health Africa Canada, 489 College Street, Toronto, ON M6G 1A5 Canada; 4 Amref Health African Tanzania, Ali Hassan Mwinyi Road, Dar es Salaam, Tanzania

**Keywords:** Gender analysis, Gender, Coverage surveys, Women’s health, Tanzania

## Abstract

**Background:**

Gender is a crucial consideration of human rights that impacts many priority maternal health outcomes. However, gender is often only reported in relation to sex-disaggregated data in health coverage surveys. Few coverage surveys to date have integrated a more expansive set of gender-related questions and indicators, especially in low- to middle-income countries that have high levels of reported gender inequality. Using various gender-sensitive indicators, we investigated the role of gender power relations within households on women’s health outcomes in Simiyu region, Tanzania.

**Methods:**

We assessed 34 questions around gender dynamics reported by men and women against 18 women’s health outcomes. We created directed acyclic graphs (DAGs) to theorize the relationship between indicators, outcomes, and sociodemographic covariates. We grouped gender variables into four categories using an established gender framework: (1) women’s decision-making, (2) household labor-sharing, (3) women’s resource access, and (4) norms/beliefs. Gender indicators that were most proximate to the health outcomes in the DAG were tested using multivariate logistic regression, adjusting for sociodemographic factors.

**Results:**

The overall percent agreement of gender-related indicators within couples was 68.6%. The lowest couple concordance was a woman’s autonomy to decide to see family/friends without permission from her husband/partner (40.1%). A number of relationships between gender-related indicators and health outcomes emerged: questions from the decision-making domain were found to play a large role in women’s health outcomes, and condoms and contraceptive outcomes had the most robust relationship with gender indicators. Women who reported being able to make their own health decisions were 1.57 times (95% CI: 1.12, 2.20) more likely to use condoms. Women who reported that they decide how many children they had also reported high contraception use (OR: 1.79, 95% CI: 1.34, 2.39). Seeking care at the health facility was also associated with women’s autonomy for making major household purchases (OR: 1.35, 95% CI: 1.13, 1.62).

**Conclusions:**

The association between decision-making and other gender domains with women’s health outcomes highlights the need for heightened attention to gender dimensions of intervention coverage in maternal health. Future studies should integrate and analyze gender-sensitive questions within coverage surveys.

## Introduction

Despite recent increases in health services, Tanzania continues to have one of the highest maternal mortality ratio (MMR) in the world (estimated at 292 per 100,000 live births in 2020, from 556 per 100,000 births in 2016) [1]. The key factors leading to maternal mortality in Tanzania include: geographic and financial access to health care, access to skilled health care workers, the quality of care received, and knowledge about sexual, reproductive, maternal and women’s health, rights, and services [2–4]. Numerous maternal health interventions have been implemented to improve maternal health outcomes, however, the MMR remains high. In order to ensure that these interventions have positive impacts on intended outcomes, it is important that they are designed and delivered appropriately. In order to do so, interventions must take into account and attempt to minimize potential barriers to access and utilization.

Coverage surveys assess whether the individuals who are in need a health care service or treatment received it, as well as other factors associated with access to health care or services [5]. They provide information on health intervention outcomes that clinicians and stakeholders can use to improve program design and implementation and evaluate their impact.

Gender is defined as the “social-constructed roles, behaviors, expressions, and identities” related to how people perceive themselves and interact with others [6]. Power relations based on gender can act as a barrier to women’s and men’s health care access and utilization by creating inequitable access to resources, roles and behaviors, norms and beliefs, and decision-making power between and among men and women [7–11]. Gender power relations may manifest as a woman’s lack of access to and control over financial and other resources [12–15], lack of autonomy and decision-making power [16, 17], restrictive gender norms [8, 18], lack of ‘women-centered’ reproductive health services [19–21], and disrespect and abuse of women by health workers during their pregnancy [22–24], all of which have been shown to impact maternal health and their health care. Additionally, there is often a lack of family planning options and health education tailored to addressing men’s fears and concerns, given norms around women being the ones primarily concerned with the family [25, 26]. While we recognize that gender is not binary, we collected data for this paper taking into account a binary lens focusing on women and men and their contextual relationships.

Ensuring that coverage surveys are gender-responsive allows researchers and implementers to understand how gender power relations may be a barrier to intervention or treatment coverage and use. By doing so, interventions may be modified and activities adapted to address these barriers, ensuring that an intervention meets its objectives to improve health and wellbeing. Gender analysis seeks to understand gender power relations and their implications on health outcomes and within health systems [11]. By focusing on power relations, it explores the causes and consequences of differences between and among men and women. It also investigates how these differ by social stratifications, including age, race, ethnicity, income, education, ability status, etc., as well as how programs, interventions, services, and policies can respond to these differences in a way that does not cause unintended consequences. Due to their complexity and context-specific nature, gender power relations can be difficult to measure and assess in a quantitative manner; in fact, this complex understanding of gender has only been operationalized in a few structured surveys [27]. Therefore, qualitative methods are often used to undertake gender analyses, which allow for an in-depth analysis of complex social phenomena, but cannot describe the quantitative prevalence of trends and risk factors.

Whether using qualitative or quantitative methodologies, it is difficult to assess gender power relations directly. Participants’ responses are often subject to recall, social desirability, and other internal biases [28]. Therefore, proxies are often used to explore how gender power relations manifest through the use of gender questions or gender analyses. For example, differential access to resources can be explored via access to income, education, time, or technology between and among men and women. Previously published frameworks for assessing gender also highlight the multi-domain approach necessary for accurately measuring these dynamics [11, 29–32], however, these have often been more limited in the health outcomes they explored.

In light of these gaps in prior research, we conducted a gender analysis of coverage survey data in Simiyu region, Tanzania from the Real Accountability: Data Analysis for Results (RADAR) project to understand whether the various domains of gender dynamics are associated with women’s health service coverage. This work was conducted in partnership with Amref Health Tanzania, which is dedicated to improving reproductive, maternal, newborn, child, and adolescent health outcomes. This study expands upon prior work to apply a broader gender analytic framework to coverage assessments [11], particularly in an area with relatively low levels of health services coverage and higher levels of gender inequity.

## Methods

### Study setting

The Simiyu region lies in north-western Tanzania in the Lake zone, made up of 5 districts and Bariadi Town is the administrative headquarters. It has an overall population of 1,584,157 people, and 93% of the area is rural [33]. The main occupation is subsistence farming and pastoralism [34]. About half of all residents are under 14 years of age (51.3%) [33]. The Lake zone has an under-5 child mortality rate of 88 deaths per 1000 live births (compared to 67 deaths per 1000 live births in Tanzania overall), and only 50% of births were delivered in a health facility (compared to 63% in all Tanzania) [35]. In the Lake zone, 51% of births were attended by skilled health personnel, which is 13% less than the average of Tanzania. In Simiyu specifically, there is lower coverage of interventions compared to national averages: for instance, 68% of children ages 12 to 23 months received all their vaccinations, while the average in Tanzania is about 75% [35].

### Study population and coverage survey

We used multi-stage cluster sampling, stratified by area of residence (urban, rural, mixed) to measure coverage within the Simiyu region. For the first stage, we used systematic random sampling with probability proportional to population size to sample 67 clusters. The 2012 Tanzania census served as sampling frame, and enumeration areas (EAs) from the census were used as clusters. Within each of these clusters, we listed and enumerated all the households, then sampled 30 households using systematic random sampling. In total, 2010 households were selected. Trained interviewers visited each sampled household and, after obtaining verbal consent, drew up a roster of all household members. Interviewers used this roster to identify all women aged 15–49 in all the sampled households and all men aged 15–49 in a randomly selected sub-sample of 1005 households (50% of the sample). The household response rate was 98.8% with 1915 household surveys completed. The women’s overall response rate was 94.1% with 2528 women included in our sample; the women’s questionnaire included questions on women’s sociodemographic information, fertility, antenatal care and childbirth, postnatal care, family planning, and gender modules. The men’s overall response rate was 87.2% with 1000 total men in our sample; the men’s questionnaire included questions about men’s sociodemographic information, family planning, and gender modules. All interviews were carried out February–April 2018 primarily in Swahili and in a few cases in Sukuma, a local language.

### Incorporating gender analysis questions into the coverage survey

We conceptualized gender power relations using Morgan et al.’s gender framework [11], which spans four major domains: (1) decision-making and autonomy, (2) labor sharing and partner involvement, (3) access to resources, and (4) norms and beliefs. Next, we developed a gender analysis matrix to identify key gender-related considerations and questions across relevant coverage survey domains (i.e. access to and utilization of services, quality of care, facility/infrastructure). Examples of these tools are accessible on the RADAR project website (www.radar-project.org) [36].

We reviewed the existing coverage survey to identify questions which could be used as proxies for gender analysis. We reviewed validated indicators (for example final reports and survey instruments used in published Demographic and Health Surveys [37] and Multiple Indicator Cluster Surveys [38]) to fill any existing gaps, prioritized questions for inclusion within the coverage survey (based on feasibility and appropriateness), and incorporated these new gender analysis indicators into the coverage survey. Questions span across the four gender analysis domains identified above. These additional variables necessitated the development of a standalone work and decision-making module within the women’s survey. We also developed a men’s questionnaire with relevant modules to complement the questions and modules within the women’s survey. Many, though not all, questions were asked in both the men’s and women’s questionnaires. The decision-making and norms/beliefs domains were most heavily represented within the survey. This was due both to the coverage survey tool’s goal of keeping the questionnaires as light as possible, and to the limited availability of validated indicators that could be used as proxies. We were unable to include additional labor sharing questions, for example, as these would require intensive time-use questions. Similarly, more comprehensive gender-based violence questions were not able to be included due to time intensiveness for training and interviewing, and the ethical requirements in the context of a household coverage survey. Further details of the questionnaire, including the specific questions used in the coverage survey, are available on the RADAR website [36].

### Conceptual model

**Fig. 1:**
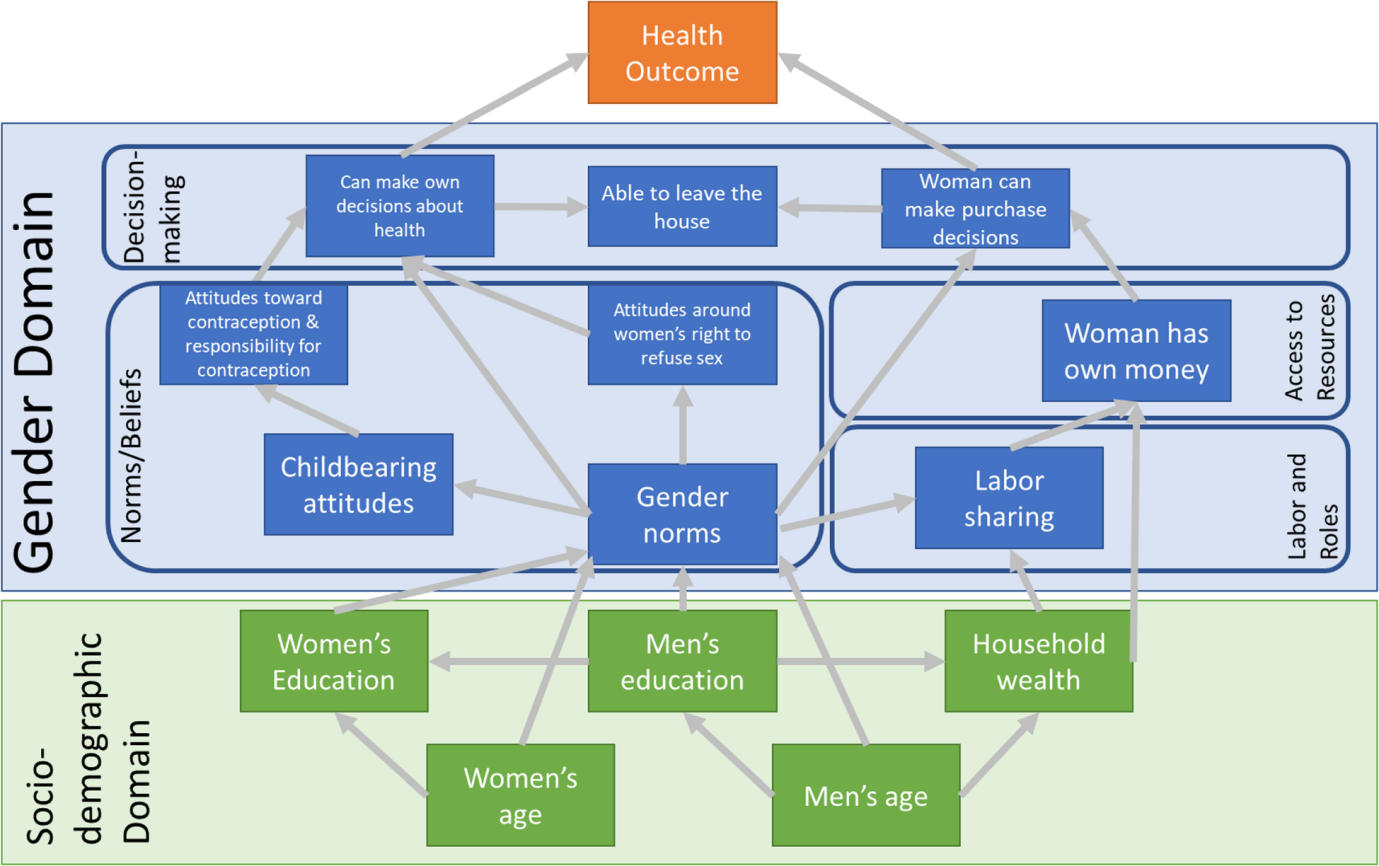
Conceptual framework with directed acyclic graph notation of gender indicator variables and their associations between one another and with health outcomes

**Fig. 2:**
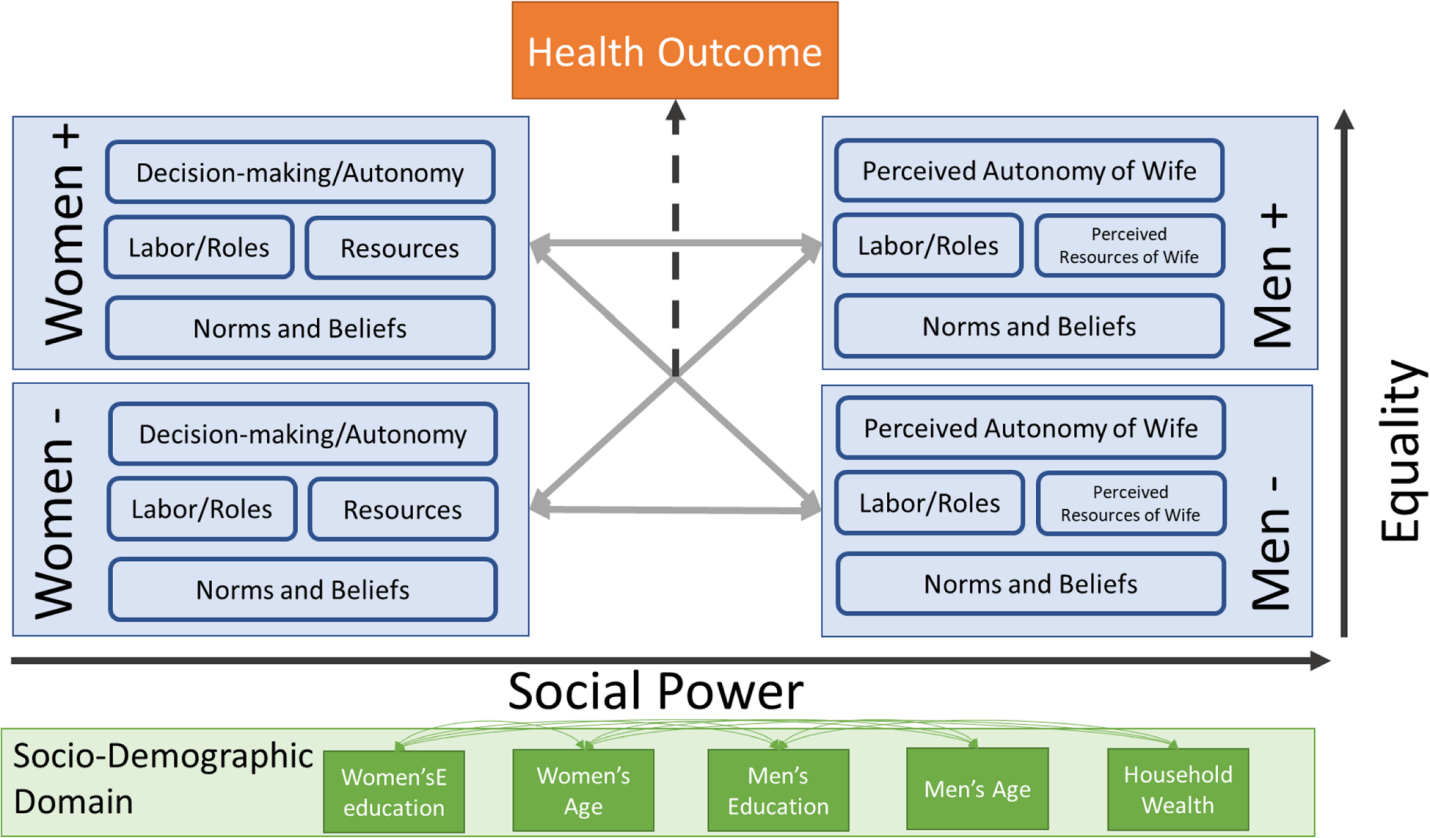
Conceptual framework of social power and equality in relationships and the impact of concordance or discordance in the relationship on health outcomes. Note: “Women +” refers to women’s endorsement of these gender variables as measured in our survey, while “Women -” refers to lack of endorsement. Similarly, “Men +” refers to men’s endorsement while “Men -” refers to their lack of endorsement of gender power dynamic variables. We hypothesize that men carry more social power in patriarchal societies than women, and that endorsing positive gender variables relates to greater equality within the relationship/marriage. We highlight here that it is the overlap and lack thereof of their gender variable endorsement (concordance or discordance of responses) that is of interest in this analysis of health outcomes

In order to map our multi-domain gender indicators to our outcomes of interest, while controlling for potential confounding due to sociodemographic variables, we used a series of conceptual models to guide our thinking (Figs. 1 and 2). Drawing upon the broader conceptual models of the previously described gender analytic framework and the socioecological framework, we positioned our sociodemographic confounders as the foundation upon which many gender is a foundation. We grouped our indicators based on the four domains and drew relationships initially based on these overall groupings. Finally, we applied directed acyclic graph (DAG) notation from our measured indicators to one another, in order to formalize the regression model we intended to run [11] (Fig. 1).

These conceptual models examined women’s gender norms and beliefs, decision-making ability, access to resources, and paid labor and responsibility-sharing in the household. We also applied an extended framework to incorporate complimentary measures in the men’s survey. Utilizing a paired analytic approach, we limited our analyses to men’s and women’s responses who could be paired from the survey’s relational data. We hypothesize that social power and equality exist on two axes that men’s and women’s concordant or discordant responses to gender variable questions can then influence health outcomes (Fig. 2). To simplify the discussion of the discordance analyses, “men discordant” refers to when the men endorsed an item and their wife/partner did not; whereas, “women discordant” refers to when the women endorse an item and their husband/partner did not.

### Major outcome variables

The gender variables were assessed against 18 existing women’s health and health system access outcome indicators, spanning three levels: individual, family, and health system. Individual outcomes included whether a woman breastfed her last child. Family outcomes included those requiring consent or input from both partners, such as whether a woman used condoms or contraception during her last sexual encounter with their partner/husband. Health system access outcomes included outcomes that were at least partially dependent on health facility capacity and availability, such as whether a woman received Antenatal Care (ANC) from a skilled health care provider. We removed health outcomes from our analyses that had poor performance in prior RADAR coverage surveys, such as whether a skilled provider was present at a woman’s most recent delivery.

### Statistical analysis

We conducted the following statistical analyses:

#### Descriptive analysis of all men and all women

We undertook initial descriptive analyses to understand the distribution of gender variables within the sample population. For this assessment, we identified men and women residing in the same household. In addition, multiple men or women from the same household could be included in the main analysis, however additional people beyond the head of household and their partner were removed in the paired analysis.

#### Descriptive analysis of paired men and women

We also undertook descriptive analysis among paired men and women who were married to one another. We restricted our analysis to men who were heads of their households and their wives/partners. This is due to data availability; in the household roster, each household member’s relationship was recorded with respect to the head of household. We calculated the percent agreement and Pearson’s correlation coefficient for each of the responses between couples. Polygamous unions were not delineated from other unions, and only the primary husband-wife pair were included in the analysis.

#### Inferential analysis

In order to conserve power, we generated 2 × 2 contingency tables for each gender variable and health outcome. If a contingency table had fewer than 10 cases in any given cell, we excluded the corresponding dependent/independent variable model from analysis. We then calculated logistic or linear regressions for each health outcome and the gender variables of interest, as applicable. We used a false discovery rate (FDR) method to adjust for multiple testing while maintaining power in items with fewer responses due to the survey skip pattern. Our intended significance threshold was defined as *P* < 0.05.

We also generated an overarching DAG to conceptualize the relationship between gender variables and our health outcomes of interest. For health outcomes that showed consistent unadjusted associations with 2 or more gender variables, we generated more detailed DAGs that used individual gender variables in order to inform adjustment in subsequent regression models. For all unadjusted models that had statistical significance, we used models adjusting for covariates identified in the DAGs, including women’s and men’s education, household wealth quintile, and women’s and men’s ages.

Sampling weights were accounted for in the survey design and for household and individual non-response. All analyses were weighted and used the Taylor linearization method to adjust standard errors for the effects of clustering and stratification. Data cleaning was conducted in Stata 14 [39]. All statistical analyses were performed using the R Statistical Software version 4.0.2 [40].

## Results

### Sociodemographic results

Across 2002 households in northern Tanzania, there were 2528 female respondents and 1000 male respondents (Table 1). The majority of households were from rural areas (weighted N [wN] = 2002, 74.0%).

**Table 1:** Descriptive statistics for sociodemographic characteristics of women and men with weighted estimates

	Women ***N*** = 2528	Men ***N*** = 1000
Age	Weighted N (%)	Weighted N (%)
< 15–19	572 (22.6%)	273 (27.4%)
20–29	935 (37.0%)	327 (32.9%)
30–39	551 (21.8%)	202 (20.3%)
40+	469 (18.5%)	192 (19.3%)
Educational Status^a^		
No education	520 (20.6%)	108 (10.8%)
Primary education	1683 (66.6%)	709 (71.3%)
Secondary education	324 (12.8%)	176 (17.7%)
Household Wealth Quintile		
Quintile 1	411 (16.3%)	131 (13.1%)
Quintile 2	495 (19.6%)	179 (17.9%)
Quintile 3	532 (21.1%)	204 (20.5%)
Quintile 4	611 (24.2%)	282 (28.3%)
Quintile 5	477 (18.9%)	198 (20.0%)
Urbanicity		
Urban/Mixed	654 (25.9%)	268 (27.0%)
Rural	1873 (74.1%)	726 (73.0%)
Married or living with partner^a^		
Not in Union	730 (28.9%)	449 (45.1%)
In Union	1796 (71.1%)	544 (54.7%)

^a^*Does not add up to the total due to some participants reporting “Don’t know”. Total number of respondents reporting “Don’t know” to major demographic questions were < 0.5%*

### Health outcomes

Health outcomes with nearly full intervention coverage (Table 2) were not included in the inferential analysis; models for gender variables regressed on these health outcomes could not be fit due to the small sample. The outcomes excluded were “*breastfeeding one’s last child*” and “*seeing a skilled provider for ANC*”. The outcome with lowest coverage were “*condom use during a woman’s last sexual encounter*” (9.9, 95% CI: 8.6–11.0%), while the highest coverage was whether a woman had “*ever breastfed her last child delivered in the past 2 years*” (97.8, 95% CI: 96.9–99.0%). Other health outcomes with variable coverage included whether a woman had “*visited a health facility for herself (as opposed to only for her family) in the past year*” (33.5, 95% CI: 31.6–35.0%) and whether “*she and her partner were currently using contraception*” (22.9, 95% CI: 20.8–25.0%).

**Table 2:** Descriptive statistics of health outcome data with weighted responses from the women’s health coverage survey

	Received health coverage outcome % (95% CI)	Weighted N^b^
Currently using condoms with partner	9.9% (8.6, 11.0%)	209
Currently using contraception with partner	22.9% (20.8, 25.0%)	457
Breastfed last child delivered in the past 2 years^a^	97.8% (96.9, 99.0%)	1162
Received iron in last pregnancy in the past 2 years^a^	70.2% (67.3, 73.0%)	833
Gave birth in health facility in last pregnancy in the past 2 years^a^	57.7% (54.6, 61.0%)	685
Went to health facility for herself in the past year	33.5% (31.6, 35.0%)	906
Saw someone for ANC in the past 2 years^a^	96.6% (95.5, 98.0%)	1148
Skilled provider for ANC in the past 2 years^a^	65.6% (27.1, 31.0%)	783
PNC visit at health facility after giving birth in the past 2 years^a^	58.8% (54.4, 63.0%)	403
Went to health facility for PNC check-up in the past 2 years^a^	86.5% (79.9, 91.0%)	131

*ANC* Antenatal care; *PNC* Post-natal care

^a^These questions were only asked among women who had given birth in the last 2 years

^b^Weighted by survey sample weights

### Gender Indicator variables

#### Autonomy and decision-making

There were a range of endorsement of autonomy/decision-making items among women in our sample (Table 3). For example, 72.3% (wN = 1958) indicated that they or both they and their husbands made decisions about how many children to have. However, only 32.2% (wN = 872) reported they could make major purchase decisions on their own or with a partner, and 39.2% (wN = 1061) reported being able to decide whether they can visit family.

**Table 3:** Descriptive Statistics for women and men who endorsed gender variables in their respective coverage surveys

	Women (Yes) wN (%)^a^	Men (Yes) wN (%)^a^
**Decision-making/ autonomy**		
Women are able to leave the house	1525 (56.3%)	504 (46.9%)
Women can make own decisions about health	1654 (61.1%)	302 (47.6%)
Women can make major purchase decisions	872 (32.2%)	244 (42.3%)
Women can make decisions to visit friends/family	1061 (39.2%)	991 (92.2%)
Women/both make daily purchases	1499 (55.4%)	627 (41.7%)
Women/both sell poultry	1480 (54.7%)	588 (54.7%)
Women/both sell livestock	829 (69.4%)	377 (35.1%)
Women/both decide how many children	1958 (72.3%)	777 (72.3%)
Women decide how to use their own money	79 (21.5%)	425 (77.0%)
**Labor sharing and roles**		
Partner accompanied to ANC	868 (76.2%)	*NA*^b^
Partner accompanied to delivery	477 (68.2%)	*NA*
Husband attended HF for family	*NA*	498 (46.3%)
**Access to resources**		
Woman has her own money	1614 (59.6%)	*NA*
Woman worked last week	1625 (60.0%)	324 (51.3%)
Woman worked last year	491 (39.5%)	97 (35.7%)
Access to mobile banking	667 (68.3%)	*NA*
Woman has own bank account	68 (2.5%)	6 (0.9%)
Has mobile phone	977 (36.1%)	*NA*
**Norms and beliefs**		
Husband justified in beating wife	2309 (85.3%)	815 (30.1%)
Childbearing is women’s concern	668 (25.2%)	226 (21.8%)
Doctor is necessary for delivery	3654 (98.6%)	1034 (97.9%)
Should the husband accompany to ANC?	994 (94.4%)	*NA*
Should the husband accompany to delivery?	975 (92.7%)	*NA*
Contraception is women’s concern	827 (31.7%)	193 (18.7%)
Contraception is for promiscuous women	266 (10.3%)	97 (9.7%)
Woman has right to refuse sex from husband	676 (25.9%)	301 (29.4%)
If woman refuses sex, husband has right to reprimand/get angry with her	1507 (57.3%)	496 (49.3%)
Husband has right to refuse money	682 (25.8%)	195 (18.9%)
Husband has right to use force for sex	683 (25.8%)	127 (12.4%)
Husband has right to have sex with another woman	703 (27.2%)	204 (20.4%)

^a^Weighted N (wN); ^b^NA” refers to “Not Asked,” as not all gender variables were asked in both the men’s and women’s survey

#### Labor-sharing and roles

The majority of women reported their partners accompanied their wife to ANC appointments (76.2%, wN = 868) and delivery in general (68.2%, wN = 477), though more attended ANC than delivery.

#### Financial resources

Among all women, 60% reported having their own money, and 60% reported working for paid income in the past week (Table 3). This is slightly higher than the men’s responses, where 51.3% of men reported their wife working for paid income in the last week. Many women responded that they have access to mobile banking (wN = 68.3%), while only a minority reported having their own mobile phone (wN = 977, 36.1%) and an even smaller number of women had their own bank account (wN = 68, 2.5%).

### Social norms and roles

The majority of women interviewed endorsed at least one situation in which a husband was justified in beating his wife (85.3%, wN = 2309). In contrast, only a small proportion of men reported the same (wN = 815, 30.1%). However, most women endorsed social norms around shared roles in family planning. For instance, 74.8% (wN = 1985) reported that childbearing was not solely a woman’s concern, and nearly all responded partners should accompany their wife to ANC visits (94.4%, wN = 994) and delivery in general (92.7%, wN = 975).

### Testing the effect of gender dynamics on health outcomes

We presented these results by gender variable to highlight the number of associations with each gender indicator variable and their potential association with health outcomes. Of the four gender domains, decision-making was found to have the strongest association with health outcomes (Table 4). We found that women making their own decisions about their health increased the odds of using condoms and contraception by over 40% (P: 0.048 and 0.013, respectively). In addition, making major household purchase decisions and daily purchase decisions were also associated with increased family health outcomes and health facility-level outcomes. Of decision-making variables, whether a woman was able to decide how to use her own money had the highest effect size (OR: 2.34, 95% CI: 1.28, 4.29), and was significantly associated with deciding to go to a health facility for her own health care.

**Table 4:** Adjusted individual gender models and outcomes with significant association (grouped by gender domain)

Gender Variables	Odds Ratio (95% CI)	P-value	Outcome variable
**Decision-making and autonomy**			
Making own decisions about health	1.42 (1.004, 2.00)	0.048	Used condoms
Making own decisions about health	1.41 (1.08,1 .84)	0.013	Used contraception
Making own decisions about health	1.28 (1.06, 1.55)	0.010	Went to HF for herself
Make major purchase decisions	1.31 (0.93, 1.83)	0.12	Used condoms
Make major purchase decisions	1.43 (1.01, 2.02)	0.044	Health worker visited
Make major purchase decisions	1.50 (1.1, 1.95)	0.0022	Used contraception
Make daily purchase decisions	1.43 (1.04, 2.04)	0.030	Used condoms
Make daily purchase decisions	1.48 (1.04, 2.10)	0.030	Health worker visited
Make daily purchase decisions	1.26 (0.97, 1.62)	0.080	Used contraception
Make daily purchase decisions	1.26 (1.05, 1.52)	0.012	Went to HF for herself
Make decisions about selling poultry	1.36 (1.06, 1.76)	0.017	Used contraception
Make decisions about selling poultry	1.36 (1.13, 1.63)	0.001	Went to HF for herself
Make decisions about selling livestock	1.18 (0.90, 1.56)	0.24	Used contraception
Make decisions about selling livestock	1.41 (1.16, 1.71)	0.00056	Went to HF for herself
Make decisions about selling livestock	3.08 (1.50, 6.31)	0.0022	Skilled postnatal check
Both decide how many children	1.52 (1.12, 2.05)	0.007	Used contraception
Both decide how many children	1.41 (1.14, 1.73)	0.0012	Went to HF for herself
Woman is able to decide how to use her own money	2.34 (1.28, 4.29)	0.0063	Went to HF for herself
**Labor-sharing and partner involvement**			
Partner attended ANC	2.81 (1.53, 5.15)	0.0009	Received tetanus
Husband attended HF for family	1.87 (1.22, 2.87)	0.0039	Health worker visited in past year
Husband attended HF for family	1.58 (1.21, 2.06)	0.00089	Went to HF for herself
**Access and resources**			
Has own money	1.63 (1.17, 2.28)	0.0040	Used condoms
Has own money	1.40 (1.10, 1.79)	0.0062	Used contraception
Has own money	1.93 (1.39, 2.68)	0.00011	Checked on at HF after giving birth
Access to mobile banking	2.08 (1.18, 3.65)	0.011	Used condoms
Access to mobile banking	1.40 (1.10, 1.79)	0.0062	Used contraception
Access to mobile banking	1.11 (0.78, 1.56)	0.56	Went to HF for herself
Woman has mobile phone	1.97 (1.40, 2.76)	0.00010	Used condoms
Woman has mobile phone	1.69 (1.29, 2.22)	0.00015	Used contraception
Woman has mobile phone	1.37 (1.11, 1.68)	0.0030	Went to HF for herself
**Social norms and beliefs**			
Woman believes husband justified is in beating	0.73 (0.43, 1.01)	0.057	Used contraception
Woman believes husband has right to refuse money	0.51 (0.33, 0.79)	0.0025	Checked on at HF
Woman believes husband has right to force sex	0.79 (0.58, 1.07)	0.13	Used contraception
Woman believes husband has right to have sex with another woman	0.62 (0.41, 0.94)	0.026	Health worker visited in last year
Woman believes husband has right to have sex with another woman	0.51 (0.33, 0.77)	0.0017	Checked on at HF

All models were adjusted for women’s education, women’s age, household wealth, and household urbanicity. *P*-values are adjusted for false discovery rate

*HF* Health facility

Access to resources and labor-sharing were the two smallest categories of gender domains considered, however, associations remained. For example, the husband attending the health facility for the family (e.g., for his wife or child, as opposed to solely for himself or not at all) was associated with a health worker visiting the family in the last year (OR: 1.87, 95% CI: 1.22, 2.87), and the woman going to the health facility for her own health care (OR: 1.58, 95% CI: 1.21, 2.06).

If a man or woman held beliefs that a husband had the right to beat his wife for various reasons (i.e., right to reprimand, refuse money, force sex, or have sex with another woman if his wife refused to have sex with him), were associated with worse health and health system outcomes. All significant associations were at the health system access level. For example, she was less likely to be have a postnatal care (PNC) appointment after giving birth if she believed he was justified in refusing money (OR: 0.51, 95% CI: 0.33, 0.79), or to have sex with another woman (OR: 0.51, 95% CI: 0.33, 0.77) if his wife refused to have sex with him.

### Paired analysis of partners in same household

We had 356 couples (head of household-wife pairs) overall we could link through relational data from the household surveys for the paired analysis. Within the paired analysis, there was statistically significant agreement for the majority of gender-related variables. The percent of agreement ranged from 40.1 to 99.2%. The lowest agreement was reported among whether women could decide to visit their family/relatives, with the majority of couples disagreeing (wN = 205, 57.9%), with most men reporting yes and most women reporting no (Table 5).

**Table 5:** Agreement and correlation statistics for paired analysis of gender variables within a family

Gender Variable	Agreement	Pearson’s Correlation Coefficient	N (%) Discordance (women “yes”, men “no”)	N (%) Discordance (women “no”, men “yes”)
**Decision-making and autonomy**				
Women is able to leave the house	63.3%	0.27***	76 (21.5%)	54 (15.3%)
Women can make own decisions about health	54.0%	0.09	107 (30.2%)	56 (15.8%)
Women can make major purchase decisions	59.3%	0.15**	40 (12.3%)	94 (28.6%)
Women can make decisions to visit friends/family	40.1%	0.03	7 (2.0%)	205 (57.9%)
Women/both sell poultry	60.7%	0.22***	85 (24.0%)	54 (15.3%)
Women/both sell livestock	72.0%	0.38***	36 (10.2%)	63 (17.8%)
Women/both decide how many children	66.7%	0.17**	48 (13.6%)	70 (19.8%)
Women decide how to use own money	48.3%	0.25	1 (1.7%)	30 (50.0%)
**Access to resources**				
Woman worked last week	68.6%	0.42**	101 (28.5%)	10 (2.8%)
Woman worked last year	72.4%	0.41**	15 (25.9%)	1 (1.7%)
Woman has own bank account	99.2%	0.67***	2 (0.6%)	1 (0.3%)
**Norms and beliefs**				
Husband justified in beating wife	74.7%	0.29***	72 (20.2%)	18 (5.1%)
Childbearing is women’s concern	72.7%	0.26***	60 (17.2%)	37 (10.6%)
Doctor is necessary for delivery	93.2%	−0.04	12 (3.4%)	12 (3.4%)
Contraception is women’s concern	72.9%	0.36***	73 (20.8%)	22 (6.3%)
Contraception is for promiscuous women	90.7%	0.26***	18 (5.3%)	14 (4.1%)
Woman has right to refuse sex from husband	70.9%	0.25	48 (13.7%)	54 (15.4%)
If woman refuses sex, husband has right to reprimand/get angry with her	57.9%	0.16	90 (26.5%)	53 (15.6%)
Husband has right to refuse money	67.8%	0.07	77 (22.3%)	34 (9.9%)
Husband has right to use force for sex	73.6%	0.26***	80 (23.0%)	12 (3.4%)
Husband has right to have sex with another woman	72.2%	0.19***	63 (18.8%)	30 (9.0%)

*Note: Gender variables are listed to the left which were asked among both the women’s and men’s survey*

* p < 0.05, ** p < 0.01, *** p < 0.005

Notable discrepancies were among social norms and beliefs when a woman refuses to have sex with her husband. There was no statistically significant correlation between men’s and women’s responses regarding whether a woman had the right to refuse sex from her husband (*p*-value = 0.25). Similarly, for whether a man could reprimand his wife if she refused to have sex with him, 27% of couples were “women discordant” (wN = 90), while 16% of couples were “men discordant” (wN = 53).

We hypothesized that concordance/discordance in a relationship could relate to health outcomes based on the social power dynamics that may be present in final decision-making within a household, and prior studies of women’s autonomy and equality being associated with health outcomes. Based on our extended conceptual model (Fig. 2), we regressed the different concordance/discordance categories on health outcomes to understand whether dual agreement was associated with health outcomes, or whether one’s partner’s particular endorsement may play a greater role.

A number of associations were found after controlling for education, age, wealth, and urbanicity (Table 6). For example, couples who both responded to that they believed that childbearing was a woman’s concern were 46% less likely (95% CI: 0.15, 0.75) to attend ANC with a skilled health care provider. Conversely, if both women and men agreed that a woman was involved in decisions to visit her (i.e., the wife’s) family, the odds of skilled ANC were increased by 85% (95% CI: 1.00, 3.42).

**Table 6:** Adjusted associations of health outcomes with concordance and discordance of wife’s/husband’s responses to gender indicator variables

	Odds Ratio (95% CI)	P-value	Outcome
Childbearing is a women’s concern			Skilled ANC
Women yes, men no	0.76 (0.42, 1.38)	0.40	
Men yes, women no	0.77 (0.31, 1.90)	0.60	
Both yes	0.34 (0.15, 0.75)	0.009	
Women/both decide to visit wife’s family			Skilled ANC
Women yes, men no	1.61 (0.84, 3.11)	0.20	
Men yes, women no	1.74 (0.90, 3.35)	0.10	
Both yes	1.85 (1.00, 3.42)	0.05	
Women/both sell livestock			Skilled post-delivery check
Women yes, men no	1.58 (0.29, 8.72)	0.60	
Men yes, women no	4.67 (1.31, 16.70)	0.018	
Both yes	3.42 (0.98, 11.90)	0.054	
Women/both make major purchases			Contraception
Women yes, men no	2.85 (1.13, 7.21)	0.028	
Men yes, women no	1.72 (0.82, 3.63)	0.20	
Both yes	1.92 (0.86, 4.28)	0.11	
Women/both sell livestock			Contraception
Women yes, men no	0.60 (0.19, 1.85)	0.40	
Men yes, women no	2.10 (0.98, 4.48)	0.056	
Both yes	1.18 (0.52, 2.68)	0.70	

All models adjusted for women’s education, women’s age, household wealth, and household urbanicity. *P*-values adjusted for false-discovery rate

## Discussion

We found that a number of health outcomes were significantly associated with gender variables after adjusting for major covariates and the false discovery rate. Within this section we discuss the main findings across the four gender domains, the importance of women’s autonomy and empowerment, potential reasons for discrepancy between the women’s and men’s surveys, and how gender analysis findings from coverage surveys can be used to improve maternal health interventions.

### Decision-making and autonomy

Variables in the decision-making domain had the most associations, both within individual and health system access outcomes. Making one’s own decisions about health, major household purchases, and daily purchases were all associated with contraception use, and a number of other health system access-related outcomes, including going to the health facility for one’s own care (as opposed to only care for family). These results suggest that women’s autonomy and independent decision-making power may significantly promote positive health behaviors.

### Social norms and beliefs

Gender norms underpin many different aspects of health-seeking behaviors and health services delivery. Not only are they difficult to measure, but due to their pervasive nature, it is often difficult to establish direct causal relationships between norms and health and health systems factors and as a result, likely reflect indirect impact on health outcomes. Harmful gender norms were significantly associated with negative health outcomes, underscoring their importance in shaping health experiences and outcomes [41].

### Access to resources

This domain was associated with access to a mobile phone and mobile banking. Access to these structures may allow women to hold money for themselves and use it for personal purchases, such as medications or fees related to childbirth or ANC. Previous studies of mobile cash transfer programs among adolescent girls and young women found increased access to resources through “survival spending,” such as for basic household necessities including food and shelter, and “self-care spending,” such as clothing or sanitary items for personal use were two major spending categories [42, 43].

Also, mobile phone access offers an expanded social network and may form a communication strategy for health care workers and facilities with women about their health [44]. For example, many intervention studies have found that text message reminders, phone calls, and mobile payment incentivization have increased retention in health services [44–47]. Further research should investigate the potential role of different types of resource access and where gendered access to resources play the most role in impacting health outcomes.

### Labor-sharing and roles

We limited our gender indicator variables to only validated metrics in a coverage survey. While labor-sharing and gender roles have been studied extensively, one of the best ways to assess the division of labor includes direct observation, which was not possible in the context of a coverage survey. Therefore, while we found few associations with labor-sharing, this area needs further research. The specific indicators we used may not correctly measure the construct of labor distribution in this context, or this may reflect a true null effect, where the other domains of gender play a larger role in health services access.

### The importance of women’s autonomy and empowerment

For analytical purposes in our inferential analyses with the women’s survey, we explored gender power relations across each of the four gender domains (decision-making, roles, norms, and access to resources) separately. However, it is important to recognize that these domains intersect and relate to one another. Gender norms, for example, influence roles and behaviors, access to resources, and decision-making power; while decision-making power influences access to resources and roles and behaviors. Due to the complexity and context-specificity of gender power relations, it is difficult to explore the interplay between the different domains quantitatively. Despite this, associations were found between the different gender domains and health and health systems outcomes, highlighting the role and importance of gender power relations in shaping health.

These findings suggest that women’s actual decision-making power in particular, may have a direct relationship on their health. Previous studies of gender dynamics and health have focused on women’s empowerment [48–51], using a range of metrics, including ecological measures of women’s involvement in parliament and economic positions [52], DHS survey questions around decision-making [27, 53, 54], as well as emic definitions and context-specific scales developed through formative research [55]. These studies have found associations between gender domains and health outcomes and highlight that gender-responsive and gender-transformative interventions can improve these outcomes [48, 51]. For example, one study from urban Nigeria found that women who had access to their own money had a 16% increase in the odds of using modern family planning methods [51]. Our study compliments such findings, and expands the number of health outcomes beyond those previously associated with gender dynamics, but also to broader health outcomes, including those at least partially controlled by health facility resources.

### Gendered discrepancies between men and women

In the paired analysis, there were a number of indicators without a statistically significant correlation between men and women within a household, suggesting noticeable discrepancies between responses. Most notably,  women’s ability to make decisions about their own health was not significantly concordant between men’s and women’s surveys, with more women reporting they could make their own health decision and men reporting that woman could not. Women reporting that they could make decisions about their own health had significant associations with improved health outcomes (while men reporting that they could make their own health decisions did not show such associations), suggesting that women’s self-reported and own understanding of autonomy is most impactful.

Other areas with low concordance were between social norms and beliefs, such as a woman having the right to refuse sex, and whether the husband has the right to reprimand her when she refuses sex. Across all of these social norms, more women than men reported that men had the right to retaliate. Further work must be done to understand this potential effect better.

These discrepancies may be due to social acceptability bias, whereby men and women are more or less likely to endorse gender norms, based on their perception of the interviewer(s) and the most acceptable response [56]. Such discrepancies may also be due to different perceptions of gender and gender equity between men and women. For example, men may not perceive women’s lack of decision-making power to be problematic due to gendered norms around men’s and women’s roles. Or they may think that the limited power that women do have is enough, so when asked if women are allowed to make decisions respond in the affirmative. Alternatively, they may think that women have a lot more power and autonomy than they do vis-à-vis the status quo. In our experience, we have found that those in positions of power are often blind to the status quo as their lived experiences are positively shaped by it, and that is their norm. In contrast, those who lack power are often more aware of it and its consequences.

### Using gender analysis results within coverage surveys to improve maternal health interventions

We found that health surveys do not often include multiple gender variables [5, 57, 58]. This is likely because it can be difficult to see how gender power relations are directly related to health outcomes. For example, a question about whether a woman has access to resources does not in itself directly relate to health outcomes – further analysis is required to connect these associations. This is because gender inequity is a social determinant of health, a root cause of health inequity. Addressing root causes requires multi-sectoral collaboration and sustained effort over the long term.

We found that gender power relations are related to health outcomes. The purpose of a coverage survey is to explore whether those in need of an intervention received it and to identify potential barriers to accessing these interventions. Understanding how gender affects health outcomes allows us to better understand how gender power relations affect an intervention’s ability to reach its target population. By doing so, we can modify our interventions according to the context to ensure that our interventions are gender accommodative (i.e., they take into account gendered considerations or barriers), or transformative (i.e., they challenge or change harmful gender norms, roles, and relations) [9]. By not considering the gendered context in which interventions are implemented, we ignore the inequities that shape people’s lived experiences and reality and potentially perpetuate and reinforce harmful gender norms, roles, and relations.

According to the findings presented here, sexual, reproductive, and maternal health interventions should seek ways to promote women’s autonomy and decision-making power if they are to improve health outcomes. Increasing access to resources, such as a mobile phone and mobile banking, can also help increase women’s autonomy and improve health outcomes. However, a gender-sensitive implementation is extremely important so as to ensure that interventions do not have unintended consequences which further marginalize women [27]. Likewise, interventions should consider whether and how males are engaged [59]. Male engagement should align with what women want and not perpetuate male decision-making power [60].

### Limitations

The study had a number of limitations. Firstly, there were time and budget constraints given that gender dynamics were not priority indicators in the parent coverage survey. We were required to use an accessible number and depth of indicators and were restricted to only previously validated measures. This resulted in a somewhat lopsided assessment, with more questions feasible in the decision-making and social norms domains, and fewer possible questions in the access to resources and labor-sharing domains. For example, validated metrics of labor-sharing include time-use surveys [61–64]. This was not possible in the context of a cross-sectional survey, and therefore only 3 questions could be asked. Additionally, the majority of the interviewers who administered the parent survey were male, which held over to the gender-specific survey as well and may potentially skew responses. For instance, women may underreport interpersonal violence measures.

In addition, analyses were limited to descriptive statistics and basic inferences in order to rigorously explore one of the first comprehensive assessments of gender dynamics in a health coverage survey. In order for reproducibility of this methodology, we used a straightforward study design. This limited the ways we could aggregate our measures, and interpretability of each individual gender dynamic and health outcome remains a challenge. Future studies will make use of more complex methods to elucidate further interactions between gender variables and other sociodemographic factors, to impact health outcomes. Finally, while these are important associative findings, we used cross-sectional data from a household coverage survey and did not undertake a formal causal analysis. Future research is needed to establish temporality and potential causal associations.

## Conclusion

Overall, we found a number of associations among gender indicators with health outcomes at the individual-, family-, and health facility-level. We recommend that gender be incorporated in other coverage surveys, including addition of a men’s questionnaire when possible*.* By integrating gender into coverage surveys and conducting gender analyses, researchers and practitioners may ensure that their interventions are not only more effective, and that they do not perpetuate and reinforce gender inequities.

## Data Availability

The data used in this study are available from the corresponding author and with approval from all institutions involved (Amref Health Africa in Tanzania and the Johns Hopkins University Institute for International Programs) upon reasonable request to the corresponding author.
